# Short-term outcomes of laparoscopic versus open liver resection for hepatocellular carcinoma in older patients: a propensity score matching analysis

**DOI:** 10.1186/s12893-022-01518-x

**Published:** 2022-02-23

**Authors:** Kazuteru Monden, Hiroshi Sadamori, Masayoshi Hioki, Satoshi Ohno, Norihisa Takakura

**Affiliations:** Department of Surgery, Fukuyama City Hospital, 5-23-1 Zao, Fukuyama, 721-8511 Japan

**Keywords:** Older patients, Laparoscopic liver resection, Propensity score matching, Short-term outcomes

## Abstract

**Background:**

The incidence of hepatocellular carcinoma (HCC) requiring surgical treatment in older patients has been continuously increasing. This study aimed to examine the safety and feasibility of performing laparoscopic liver resection (LLR) versus open liver resection (OLR) for HCC in older patients at a Japanese institution.

**Methods:**

Between January 2010 and June 2021, 133 and 145 older patients (aged ≥ 70 years) who were diagnosed with HCC underwent LLR and OLR, respectively. Propensity score matching (PSM) analysis with covariates of baseline characteristics was performed. The intraoperative and postoperative data were evaluated in both groups.

**Results:**

After PSM, 75 patients each for LLR and OLR were selected and the data compared. No significant differences in demographic characteristics, clinical data, and operative times were observed between the groups, although less than 10% of cases in each group underwent a major resection. Blood loss (OLR: 370 mL, LLR: 50 mL; *P* < 0.001) was lower, and the length of postoperative hospital stay (OLR: 12 days, LLR: 7 days; *P* < 0.001) and time to start of oral intake (OLR: 2 days, LLR: 1 day; *P* < 0.001) were shorter in the LLR group than in the OLR group. The incidence of complications ≥ Clavien–Dindo class IIIa was similar between the two groups.

**Conclusions:**

LLR, especially minor resections, is safely performed and feasible for selected older patients with HCC.

## Background

Hepatocellular carcinoma (HCC) is one of the leading causes of cancer death worldwide [1]. The risk of developing HCC depends on the epidemiology of chronic viral hepatitis (type B and C), alcohol abuse, non-alcoholic steatohepatitis, autoimmune hepatitis, and aflatoxin exposure [2]. Therefore, the currently recommended treatments, which include surgical resection, radiofrequency ablation, and liver transplantation, are considered curative treatments for HCC.

Since the first laparoscopic liver resection (LLR) was reported in 1992, LLR has been increasingly reported as an option for surgical resection, and its application has increased worldwide [3]. Several studies have reported that LLR results in shorter hospital stays, reduced blood loss, fewer complications, and earlier postoperative recovery than open liver resection (OLR) [4–7]. Furthermore, a meta-analysis of comparative studies has shown favorable short-term and long-term survival in LLR [5].

The risk of developing HCC is age-dependent [8]; therefore, with increasing life expectancy, the number of older patients with HCC is expected to increase accordingly, and the treatment for older patients remains a global issue. Compared with younger patients, older patients who may already be prescribed numerous medications may present with more comorbidities, such as chronic hypertension, diabetes mellitus, rhythm disturbance or stroke, renal insufficiency, pulmonary disease, and cancer history. Other age-related changes indicate a decline in liver and respiratory functions. Portal blood flow decreases in 30–40% of patients aged > 71 years [9, 10], whereas liver volume decreases with age in 20–40% of patients [9, 11]. Therefore, older patients are more likely to have occlusive events, and their operative course needs closer attention than those of younger patients. Nonetheless, several reports have indicated that OLR for older patients with HCC is safe and feasible [12–14]. Conversely, the benefits of laparoscopic surgery for older patients with HCC remain controversial.

Limited studies have been conducted to evaluate the risk of surgical complications or the safety and efficacy of LLR for HCC in older patients [14–16]. Thus, this study aimed to examine the safety and feasibility of LLR for HCC in older patients using propensity score matching (PSM) analysis to reduce the effect of treatment or patient selection bias.

## Methods

### Patient selection and study protocol

Initially, 290 patients aged ≥ 70 years newly diagnosed with HCC who underwent LLR and OLR in our department between January 2010 and June 2021 were enrolled in the study. Patient data were retrieved from prospectively maintained databases. Four patients were excluded from the analysis because of a history of hybrid laparoscopic surgery, and eight underwent combined procedures, including venous reconstruction and biliary reconstruction. Finally, the data on the remaining 278 patients were evaluated. Tumor-related variables, including tumor location, maximum tumor size, vascular invasion, tumor thrombus, tumor number, presence of ascites, lymph node metastasis, and extrahepatic metastases, were evaluated using imaging techniques, such as ultrasonography, multidetector-row computed tomography (CT), and dynamic contrast-enhanced magnetic resonance imaging. HCC was diagnosed in all patients based on pathology after hepatectomy. None of the patients received preoperative chemotherapy or postoperative adjuvant therapy before recurrence.

The indication for and extent of liver resection were determined as follows: tumor number ≤ 4, general condition with a performance status of 0–2, and Child–Pugh classification A or B. Indocyanine green retention rate at 15 min (ICG-R15) was used for decision-making regarding the liver resection volume. The estimated liver resection volume and future remnant liver volume were calculated using CT. If the remnant liver volume < 30% of the total liver volume, liver resection was not performed. The exclusion criteria for LLR were as follows: HCC tumor size > 10 cm, HCC with the appearance of tumor thrombus in the main portal and main hepatic veins, or HCC indicated for liver resection of more than three contiguous segments. The selection criteria for the laparoscopic approach were dependent on the surgeon’s judgment.

The Institutional Review Board of Fukuyama City Hospital (No. 452) approved this study and waived the need for informed consent from the enrolled patients. All procedures in this study were performed in accordance with relevant guidelines and regulations.

### Operative technique for laparoscopic liver resection

LLR was performed with the patient in the supine position for tumor location in the left lobe and left hemilateral position for tumor location in the right lobe. Pneumoperitoneum pressure was maintained with carbon dioxide gas kept at approximately 10 mmHg. All patients underwent intraoperative ultrasonography (IOUS) to clarify the locations of tumors and vascular structures. A 10-mm flexible camera was placed above the umbilicus, and 2–3 additional trocars, sized 5 or 12 mm, were placed in each case. Parenchymal dissection was performed using ultrasonic coagulating shears and a Cavitron ultrasonic surgical aspirator (Clarity Ultrasonic Surgical Aspirator System [CUSA®], Integra LifeSciences NR Ireland Limited, Dublin, Ireland). Hepatic veins and Glissonean branches with diameters > 3 mm were occluded using a titanium clip, and the major hepatic veins or hilar structures were divided by vascular stapling techniques. Temporary vascular inflow occlusion (Pringle maneuver) was applied during parenchymal dissection, depending on the situation, in each case. The specimen was extracted through a small median incision with a plastic bag. A local drainage tube was placed during the operation and removed within 48–72 h.

### Operative technique for open liver resection

For OLR, the patient was placed in the supine position, and the standard skin incision of an upward midline or reversed L-shaped laparotomy was made. Similar to LLR, IOUS was routinely implemented, and hepatic parenchymal dissection was performed using ultrasonic coagulating shears and CUSA with the Pringle maneuver. Vessels with diameters > 3 mm were ligated or sutured. An abdominal drain was placed during surgery and removed within 48–72 h. All surgical procedures were performed by experienced surgeons or by surgeons who were supervised by experienced surgeons.

### Data collection

We evaluated the following patient demographic data: age, sex, body mass index (BMI), history of upper abdominal surgery (i.e., cholecystectomy, gastrectomy, or previous liver resection), American Society of Anesthesiologists (ASA) physical status classification, comorbid diseases (i.e., diabetes mellitus, hypertension, cardiovascular disease, respiratory disease, and renal failure), and surgical procedure (i.e., right-sided or left-sided hepatectomy; detailed surgical techniques, such as limited liver resection, lateral sectionectomy, segmentectomy, sectionectomy, etc.). We also collected data on any medication history for antiplatelet therapy and aspirin prescription for the primary and secondary prevention of thromboembolic morbidity in the year prior to surgery. In general, with increasing age, older people are more likely to be prescribed antiplatelet therapy. In addition, our institution has continued aspirin therapy perioperatively in patients undergoing liver resection to reduce thrombotic morbidity and has collected morbidity data on postoperative hemorrhage complications.

Blood samples were collected to evaluate operative liver function by measuring the following parameters: aspartate aminotransferase, alanine aminotransferase, serum platelet count, prothrombin time, serum albumin concentration, total bilirubin, protein induced by vitamin K deficiency, alpha-fetoprotein, ICG-R15, and Child–Pugh score.

Perioperative data, including operative time, blood loss, blood transfusion rate, time to start of oral intake, length of hospital stay, morbidity, and mortality, were evaluated. To classify the degree of chronic hepatitis, the degree of liver fibrosis and necroinflammation were classified based on the New Inuyama classification [17]. Status of resection margins was classified as R0 (complete resection/no residual tumor) or R1 (microscopic residual cancer at the resection margin). The criteria for hospital discharge were absence of fever (body temperature > 37.0 °C), tolerance of oral intake, and adequate pain control over 2 consecutive days. Patients who were assessed as needing rehabilitation were transferred to the logistical support hospital.

Surgical complications within 30 days postoperatively were defined according to the Clavien–Dindo classification, which defines major complications as those graded ≥ IIIa [18]. Clinicopathological staging was determined based on the tumor/node/metastasis classification.

### Statistical analyses

#### Propensity score matching

As this study was not randomized for surgical procedures between LLR and OLR, comparing both groups on potential confounding variables was necessary. Therefore, we used PSM with a multivariate logistic regression model. The covariates included in this model were age, sex, BMI, history of abdominal surgery, comorbid diseases, history of aspirin prescription, ASA classification, hepatitis status, Child–Pugh classification, maximum tumor size, preoperative blood test, and surgical procedures (Table 1).

**Table 1:** Demographic and clinical data of patients before and after propensity score matching

	Before matching			After matching		
	OLR (n = 145)	LLR (n = 133)	*P*	OLR (n = 75)	LLR (n = 75)	*P*
Age, years	76 (70–90)	75 (70–85)	0.393	75 (70–90)	75 (70–83)	0.649
Male sex	101 (69.7)	98 (73.7)	0.457	51 (68.0)	53 (70.7)	0.723
BMI, kg/m^2^	22.6 (15.3–32.0)	23.1 (16.4–34.5)	0.4	23.0 (15.3–29.9)	23.1 (16.6–34.5)	0.891
History of open abdominal surgery, n (%)	35 (24.1)	16 (12.0)	0.008	14 (18.7)	11 (14.7)	0.511
Preexisting medical condition						
Hypertension	78 (53.8)	84 (63.2)	0.114	46 (61.3)	43 (57.3)	0.618
Diabetes mellitus	49 (33.8)	54 (40.6)	0.24	28 (37.3)	28 (37.3)	1.00
Renal failure	12 (8.3)	9 (6.8)	0.634	5 (6.7)	7 (9.3)	0.547
Ischemic heart disease	31 (21.4)	18 (13.5)	0.085	12 (16.0)	15 (20.0)	0.524
COPD or pulmonary disease	10 (6.9)	9 (6.8)	0.966	5 (6.7)	6 (8.0)	0.754
Antiplatelet (aspirin) intake, n (%)	19 (13.1)	20 (15.4)	0.643	10 (13.3)	9 (12.0)	0.806
ASA classification			0.022			0.867
I	0 (0)	0 (0)		0 (0)	0 (0)	
II	81 (55.7)	92 (69.2)		46 (61.3)	45 (60.0)	
III	64 (44.2)	41 (30.8)		29 (38.7)	30 (40.0)	
Hepatitis status			0.07			0.569
HBV positive	20 (13.8)	34 (25.5)		14 (18.7)	15 (20.0)	
HCV positive	77 (53.1)	56 (42.1)		42 (56.0)	34 (45.3)	
HBV HCV positive	2 (1.4)	3 (2.3)		1 (1.3)	1 (1.3)	
HBV HCV negative	46 (31.7)	40 (30.1)		18 (24.0)	25 (33.4)	
Child–Pugh classification			0.662			0.699
A	138 (95.2)	128 (96.2)		72 (96.0)	71 (94.7)	
B	7 (4.8)	5 (3.8)		3 (4.0)	4 (5.3)	
AFP level (ng/mL)	10 (2.0–113,560)	6 (2.0–8900)	0.069	10 (2.0–5359)	8 (2–8900)	0.953
PIVKA-II level (mAU/mL)	103 (10–115,454)	48 (2.8–24,892)	0.041	51 (10–37,080)	56 (10–24,892)	0.451
PLT (× 10^4^/μL)	14.4 (3.2–47.4)	15.6 (4–36)	0.056	14.7 (4.1–47.4)	14.6 (6.8–33.1)	0.638
ALB (g/L)	3.9 (2.8–4.8)	4.2 (2.8–5.1)	< 0.001	4.0 (2.8–4.8)	4.1 (2.8–5.0)	0.596
T-Bil (mg/L)	0.7 (0.2–2.2)	0.7 (0.2–1.9)	0.326	0.7 (0.3–2.2)	0.6 (0.2–1.9)	0.996
ALT (IU/L)	26 (6–175)	24 (2–227)	0.458	27 (9–175)	30 (2–101)	0.699
AST (IU/L)	32 (12–327)	30 (13–177)	0.149	33 (15–327)	30 (14–94)	0.777
Prothrombin time (%)	87 (60–100)	91 (68–100)	0.008	88 (66–100)	88 (68–100)	0.909
ICG-R15 (%)	13.9 (3.2–35.4)	13.0 (3.6–35.3)	0.071	13.0 (3.2–33.4)	14.4 (3.6–35.3)	0.578
Size of the largest tumor (mm)	27.0 (1.6–170)	23.0 (1.4–82)	0.012	21.0 (2.7–80)	24.0 (10–82)	0.239
Number of tumors	1 (1–4)	1 (1–3)	0.623	1 (1–4)	1 (1–3)	0.666
Surgical procedure, n (%)			0.319			0.726
Right-sided hepatectomy (excision of any segment from segment 5–8)	105 (72.4)	89 (66.9)		52 (69.3)	50 (66.7)	
Left-sided hepatectomy (excision of any segment from segment 2–4)	40 (27.6)	44 (33.1)		23 (30.7)	25 (33.3)	
Surgical procedure, n (%) (detailed surgical techniques)			< 0.001			0.961
Limited liver resection or lateral sectionectomy	50 (34.5)	69 (51.9)		35 (46.7)	37 (49.3)	
Segmentectomy	29 (20.0)	34 (25.6)		18 (24.0)	17 (22.7)	
Sectionectomy	34 (23.4)	18 (13.5)		16 (21.3)	14 (18.7)	
Hemihepatectomy or more than three sectionectomy	32 (22.1)	12 (9.0)		6 (8.0)	7 (9.3)	

Data are presented as number (%) or median (range)

*AFP* alpha-fetoprotein, *ALB* albumin, *ALT* alanine aminotransferase, *ASA* American Society of Anesthesiologists, *ASA I* normal healthy patient, *ASA II* patient with mild systemic disease, *ASA III* patient with severe systemic disease that is not incapacitating, *AST* aspartate aminotransferase, *BMI* body mass index, *HBV* hepatitis B virus, *HCV* hepatitis C virus, *ICG-R15* indocyanine green retention rate at 15 min, *LLR* laparoscopic liver resection, *N/A* not applicable, *OLR* open liver resection, *PIVKA-II* protein induced by vitamin K absence, *PLT* platelets, *T-Bil* total bilirubin

These covariates were chosen because they were previously used in other similar studies [15, 19, 20] due to their clinical relevance. The nearest-neighbor matching algorithm was employed to form pairs of patients undergoing LLR and OLR to mitigate the potential for selection bias across surgical approaches. One-to-one case matching was performed with a caliper width of 0.2 of the standard deviation of the logit of the propensity score.

Statistical analyses were performed using JMP software version 14 (SAS Institute, Inc., Cary, NC, USA). Continuous variables before matching were expressed as median (range), whereas categorical data were expressed as numbers or frequencies (%). Comparisons between the two groups were conducted using the Mann–Whitney U test. Differences in categorical outcomes were analyzed using the chi-square test, Yates’ test*,* Poisson distribution analysis, or Fisher’s exact test. Post-matching variables between the groups were assessed using the paired t-test or Mann–Whitney U test for continuous variables and the McNemar test for categorical variables. The statistical significance level was set at 0.05.

## Results

### Patient characteristics

The baseline characteristics of patients are summarized in Table 1. After PSM with a multivariate logistic regression model, 75 patients were selected for each of LLR and OLR. The groups were well matched for all covariates in this model. No significant differences in baseline characteristics were noted between the groups after PSM. Less than 10% of cases in each group had undergone a hemihepatectomy or more than three sectionectomies.

### Operative variables

After PSM, the LLR group exhibited a significantly shorter hospital stay (OLR: 12 days; LLR: 7 days; *P* < 0.001), lower blood loss (OLR: 370 mL; LLR: 50 mL; *P* < 0.001; Table 2), and shorter time to start of oral intake (OLR: 2 days, LLR: 1 day; *P* < 0.001). Furthermore, the blood transfusion rate was lower in the LLR group than in the OLR group (OLR: 13.3%; LLR: 1.3%; *P* = 0.005). No significant difference in the operative time was observed between the groups (OLR: 223 min; LLR: 263 min; *P* = 0.055). In addition, histological data, including histology and background liver characteristics, were similar between the two groups. None of the 75 patients in the LLR group needed conversion to open surgery.

**Table 2:** Operative parameters of patients in matched cohorts of laparoscopic and open liver resection

	OLR (n = 75)	LLR (n = 75)	*P*
Operative time (min)	223 (129–562)	263 (100–486)	0.055
Conversion, n (%)		0 (0)	N/A
Blood loss (mL)	370 (10–1944)	50 (0–650)	< 0.001
Blood transfusion rate, n (%)	10 (13.3)	1 (1.3)	0.005
Duration of drainage tube (days)	2 (0–28)	2 (0–18)	0.765
Time to start oral intake (days)	2 (1–8)	1 (1–7)	< 0.001
Length of hospital stay (days)	12 (6–84)	7 (4–55)	< 0.001
Histology			0.124
Well differentiated	40 (53.3)	35 (46.7)	
Moderately differentiated	27 (36.0)	37 (49.3)	
Poorly differentiated	8 (10.7)	3 (4.0)	
Microvascular invasion, n (%)	10 (13.3)	7 (9.3)	0.44
Background liver characteristics			
F3–F4	31 (41.3)	35 (46.7)	0.511
A2	28 (37.3)	26 (34.7)	0.734
Resection status, n (%)			0.477
R0	73 (97.3)	75 (100)	
R1	2 (2.7)	0 (0)	
UICC TNM classification			0.453
Stage IA	31 (41.3)	25 (33.3)	
Stage IB	26 (34.6)	28 (37.3)	
Stage II	8 (10.7)	11 (14.7)	
Stage IIIA	8 (10.7)	11 (14.7)	
Stage IIIB	2 (2.7)	0 (0)	

Data are presented as number (%) or median (range). The grade of fibrosis was classified based on the New Inuyama classification: F3: bridging fibrosis plus lobular distortion; F4: liver cirrhosis. The grade of necroinflammation was classified as follows: A2: moderate

*N/A* not applicable, *OLR* open liver resection, *LLR* laparoscopic liver resection, *UICC* Union for International Cancer Control

### Postoperative complications in the OLR and LLR groups

Postoperative complications are summarized in Table 3. After PSM, the rates of postoperative Clavien–Dindo IIIa–V complications did not differ significantly between the OLR and LLR groups (*P* > 0.05).

**Table 3:** Postoperative outcomes of patients in matched cohorts of laparoscopic and open liver resection

	OLR (n = 75)	LLR (n = 75)	*P*
Clavien–Dindo IIIa–V complications, n (%)	4 (5.3)	5 (6.7)	0.731
Respiratory failure	0 (0)	1 (1.3)	
Bile leakage	2 (2.7)	2 (2.7)	
Organ/space SSI	0 (0)	1 (1.3)	
Necrotizing fasciitis	1 (1.3)	0 (0)	
Hemorrhage	1 (1.3)	1 (1.3)	
Liver failure by ISGLS criteria > grade C	0 (0)	0 (0)	
90-day mortality, n (%)	1 (1.3)	0 (0)	1.00

Data are presented as number (%) or median (range)

*ISGLS* International Study Group for Liver Surgery, *SSI* surgical site infection, *N/A* not applicable

One patient in the LLR group experienced hemorrhage after receiving an anticoagulant, a coagulation factor X inhibitor, for cardiovascular disease (ischemic heart disease, atrial fibrillation, and installed pacemaker) early in the postoperative period. However, the patient did not undergo re-laparotomy and recovered after receiving conservative treatment. In addition, one patient in the LLR group developed respiratory failure (aspiration pneumonia).

No significant differences were noted in the 90-day mortality rates between the two groups (OLR group: 1.3%; LLR group: 0%). However, one patient in the OLR group died from necrotizing fasciitis after contracting an *Aeromonas hydrophila* infection, which commonly occurs in immunocompromised patients with hepatobiliary disease. Despite repeated surgical debridement and intensive medical treatment, he died of multiple organ failure 7 days after the initial operation.

## Discussion

In the present study, we found no significant differences in the overall postoperative complications between patients included in the LLR and OLR groups. Furthermore, postoperative parameters improved more significantly after LLR than after OLR. Particularly, blood loss was lower, and the length of postoperative hospital stay and time to start oral intake were shorter after LLR than OLR. These results suggest that LLR for HCC may result in perioperative outcomes superior to those of OLR in older patients.

We found that postoperative complications did not vary significantly between the two groups. We also found low overall complications, regardless of high comorbidities (20% of the patients with cardiovascular disease and high ASA classification), which were in accordance with recently published data [20, 21]. One of the advantages of laparoscopy is lower blood loss. Older patients have less reserve capability to compensate for circulatory disturbance due to arteriosclerosis and ischemic heart disease [22, 23]. In addition, older patients tend to be more susceptible to fluid overload and require close monitoring of their fluid balance. In case of excessive bleeding, fluid replacement must correspond to the amount of blood loss, and minimizing postoperative bleeding would be more important for older patients in maintaining respiratory and circulatory dynamics.

Additionally, we found that participants in the LLR group started oral intake earlier than those in the OLR group, indicating faster bowel function recovery after LLR. A small surgical incision may lead to decreased postoperative pain and early postoperative bowel function recovery [6]. Older patients may experience a decline in physical strength due to generalized muscle weakness caused by hypercatabolism after surgery [24] and may eventually lose their swallowing function, resulting in dysphagia. We believe that eating early and shortening the fasting period as much as possible will improve intestinal peristalsis and immune function in the intestinal tract, promote early postoperative recovery, and lead to early hospital discharge.

In our study, 19 patients in both groups were prescribed aspirin for the secondary prevention of ischemic stroke and cardiovascular disease. None of them underwent re-laparotomy or experienced postoperative hemorrhage. We previously reported that continuing aspirin medication is safe and does not increase the risk of serious hemorrhagic complications after surgery [25–27]. Furthermore, even in older patients, OLR and LLR were safely performed without interruption of aspirin therapy. These results could be attributed to secure improvements in surgical technique, surgical equipment, and careful follow-up examination for postoperative management.

Pneumoperitoneum pressure (PPP) and lower central venous pressure (CVP) in laparoscopy aid in controlling backflow bleeding from the hepatic vein [28]. In contrast, chronic obstructive pulmonary disease (COPD), which often develops in older patients, poses a higher risk for postoperative respiratory complications. In patients with COPD, airway obstruction increases intrathoracic pressure [29]. Furthermore, laparoscopic pneumoperitoneum exacerbates CVP elevation in patients with COPD. A recent study in piglets revealed that bleeding from the hepatic vein cannot be controlled by increasing the PPP under high airway and/or intrathoracic pressure [29]. Therefore, the indication for LLR in older patients with COPD should be carefully considered. Furthermore, high PPP with LLR is a risk factor associated with higher rates of pulmonary carbon dioxide gas embolism, which may induce adverse respiratory and cardiovascular events [30, 31].

This study has some limitations. First, a bias in the surgical technique was identified. The percentage of major hepatic resection (i.e., hemihepatectomy or more than three sectionectomies) was lower before and after PSM than in previous reports [20, 22]. Furthermore, the percentage of major hepatic resections was less than 10% in both OLR and LLR after PSM. Consequently, not all of the indications were applicable in this study. Second, this study had a relatively small sample size, retrospective study design, single-center design, and lack of long-term follow-up results. Lastly, we used PSM analysis, which might have overlooked unmeasured confounding factors. In addition, regarding the PSM, one-to-one PSM was used in large samples, which may create bias in the results, considering the small sample size of our study [32]. While a randomized controlled trial (RCT) would be considered less biased than observational studies, the high risk of postoperative morbidities in older patients may limit their inclusion in a RCT.

## Conclusions

We demonstrated that compared with OLR, minor LLR for HCC in selected older patients was safely performed and effective for achieving bowel function recovery, decreasing the length of hospital stay, and reducing blood loss, without the risk of increasing intraoperative or postoperative morbidity. The indication of each patient for surgery should be carefully assessed in consideration of comorbidities to avoid postoperative complications. Further investigation is necessary to assess the long-term outcomes of LLR in older patients.

## Data Availability

The datasets generated during and/or analyzed during the current study are available from the corresponding author upon reasonable request.

## Abbreviations

ASA: American Society of Anesthesiologists
BMI: Body mass index
COPD: Chronic obstructive pulmonary disease
CT: Computed tomography
CUSA: Cavitron ultrasonic surgical aspirator
CVP: Central venous pressure
HCC: Hepatocellular carcinoma
ICG-R15: Indocyanine green retention rate at 15 min
IOUS: Intraoperative ultrasonography
LLR: Laparoscopic liver resection
OLR: Open liver resection
PSM: Propensity score matching
